# Beyond the default colon: Effective use of quotes in qualitative research

**DOI:** 10.1007/s40037-019-00550-7

**Published:** 2019-11-22

**Authors:** Lorelei Lingard

**Affiliations:** Centre for Education Research & Innovation and Department of Medicine, Health Sciences Addition, Schulich School of Medicine & Dentistry, Western University, London, Canada

In the Writer’s Craft section we offer simple tips to improve your writing in one of three areas: Energy, Clarity and Persuasiveness. Each entry focuses on a key writing feature or strategy, illustrates how it commonly goes wrong, teaches the grammatical underpinnings necessary to understand it and offers suggestions to wield it effectively. We encourage readers to share comments on or suggestions for this section on Twitter, using the hashtag: #how’syourwriting?

Last week the ‘e’ key died on my laptop. It’s a first-world problem, I’ll admit, but it really threw my writing for a loop—a lot of words require an ‘e’ key. Reflecting on what other keys I could not do without, I made a quick shortlist: comma, ‘ly’ and colon. The comma because its absence would consign me to the sort of breathy, adolescent writing that fills social media. The ‘ly’ because without that duo I can’t make most of the adverbs that prop up my first drafts. And the colon because I’m a qualitative researcher. How would I introduce quotes if the colon key were out of order?

I’m only partly joking. Every qualitative researcher confronts the challenge of selecting the right quotes and integrating them effectively into their manuscripts. As writers, we are all guilty of resorting to the default colon as an easy way to tuck quotes into our sentences; as readers, we have all suffered through papers that read like a laundry list of quotes rather than a story about what the writer learned. This Writer’s Craft instalment offers suggestions to help you choose the right quotes and integrate them with coherence and style, following the principles of authenticity and argument.

## Authenticity

At the point of manuscript writing, a qualitative researcher is swimming in a sea of data. Innumerable transcript excerpts have been copied and pasted into data analysis software or (for the more tactile among us) onto multi-coloured sticky notes. Some of these excerpts we like very much. However, very few of them will make it into the final manuscript, particularly if we are writing for publication in a health research or medical education journal, with their 3000–4000 word limits.

Selecting the best quotes from among these cherished excerpts is harder than it looks. We should be guided by the principle of authenticity: does the quote offer readers first hand access to dominant patterns in the data? There are three parts to selecting a good, authentic quote: the quote is illustrative of the point the writer is making about the data, it is reasonably succinct, and it is representative of the patterns in data. Consider this quote, introduced with a short phrase to orient the reader:

*Rather than feeling they were changing identities as they went through their training, medical students described the experience of accumulating and reconciling multiple identities: ‘the “life me”, who I was when I started this, is still here, but now there’s also, like, a “scientific me” as well as a sort of “doctor me”. And I’m trying to be all of that’ (S15)*.

This quote is illustrative, providing an explicit example of the point that student identity is multiplying as training unfolds. It is succinct, expressing efficiently what other participants took pages to describe. And it is representative, remaining faithful to the overall sentiments of the many participants reporting this idea.

We have all read—and written!—drafts in which the quoted material does not reflect these characteristics. The remainder of this section addresses these recurring problems.

### Is the quote illustrative?

A common challenge is the quote that illustrates the writer’s point implicitly, but not explicitly. Consider this example:

*Medical students are undergoing a process of identity-negotiation: we’re ‘learning so much all the time, and some of it is the science stuff and some of it is professional or, like, practical ethical things, and we have to figure all that out’ (S2).*

For this quote to serve as evidence for the point of identity-negotiation, the reader must infer that ‘figure all that out’ is a reference to this process. But readers may read their own meaning into decontextualized transcript extracts. Explicit is better, even if it sacrifices succinctness. In fact, this is the right quote, but we had trimmed away the first three sentences where ‘figuring out identity’ got explicit mention. The quote could be lengthened to include these sentences, or, to preserve succinctness, just that quoted phrase can be inserted into the introduction to the quote:

*Medical students are ‘figuring out identity’, a process of negotiation in which they are ‘learning so much all the time, and some of it is the science stuff and some of it is professional or, like, practical ethical things, and we have to figure all that out’ (S2).*

### Is the quote succinct?

Interview transcripts are characterized by meandering and elliptical or incomplete speech. Therefore, you can search diligently and still come up with a 200-word quote to illustrate your 10-word point. Sometimes the long quote is perfect and you should include it. Often, however, you need to tighten it up. By including succinctness as part of the authenticity principle, my aim is to remind writers to explicitly consider whether their tightening up retains the gist of the quote.

The previous example illustrates one tightening technique: extract key phrases and integrate them into your own, introductory sentence to the quote. Another solution is to use the ellipsis to signal that you have cut part of the quote out:

*Identity formation in the clinical environment is also influenced by materials and tools, ‘all this stuff you’ve never used before … you don’t know where it is or how to use it, and don’t even get me started on the computerized record. … So many hours and I’m still confused, am I ever going to know where to enter things?’ (S7)*.

The first ellipsis signals that something mid-sentence has been removed. In this case, this missing material was an elaboration of ‘all this stuff’ that mentioned other details not relevant to the point being made. The second ellipsis follows a period, and therefore signals that at least one sentence has been removed and perhaps more. When using an ellipsis, only remove material that is irrelevant to the meaning of the quote, not relevant material that importantly nuances the meaning of the quote. The goal is not a bricolage which cuts and pastes tiny bits so that participants say what you want them to; it is a succinct-enough representation that remains faithful to the participant’s intended meaning.

Changing the wording of a quotation always risks violating the authenticity principle, so writers must do it thoughtfully. Two other situations, however, may call for this approach: to maintain the grammatical integrity of your sentence and to tidy up oral speech^1^. The first is usually not problematic, particularly if you are altering for consistent tense or for agreement of verb and subject or pronoun and antecedent, or replacing a pronoun with its referent. Square brackets signal such changes:

*Participants from the community hospital setting, however, ‘[challenged] the assumption of anonymity when evaluating teachers’. (verb tense changed from present to past)*

The second situation can be trickier: when should you tidy up the messiness of conversational discourse? Interview transcripts are replete with what linguists refer to as ‘fillers’ or ‘hesitation markers’, sounds and words such as ‘ah/uh/um/like/you know/right’ [1]. There is general agreement among qualitative scholars that quotes should be presented verbatim as much as possible, and those engaged in discourse and narrative analysis will necessarily analyze such hesitations as part of the meaning. In other applied social research methodologies, however, writers might do some ‘light tidying up’ both for readability and for ethical reasons, as long as they do not undermine authenticity in doing so [2]. Ethical issues include the desire not to do a disservice to participants by representing the um’s and ah’s of their natural speech, and the concern to protect participant anonymity by removing identifiable linguistic features such as regional or accented speech.

Finally, an emerging strategy for succinctness is to put the quotes into a table. Many qualitative researchers resent the constraints of the table format as an incursion from the quantitative realm. However, used thoughtfully, it can offer a means of presenting complex results efficiently. In this example, Goldszmidt et al. name, define and illustrate five main types of supervisor interruptions that they observed during their study of case review on internal medicine teaching teams (Tab. 1; [3]).

**Table 1:** Types of supervisors’ interruptions during patient case review presentations, London Health Sciences Centre, University Hospital, Ontario, Canada 2010

Type	Description	Example
Probing for further data	Supervisors ask questions about patient facts, management details, or clarification	Case 17; AM CC-5: Her haemoglobin was 94. A‑9: Do we have a previous? CC-5: Yeah, she had one done at the cancer clinic
Prompting for expected sequence	Supervisors indicate what is expected to come next in the presentation, either proactively or as a correction	Case 10; AM A‑3: Cardiovascular exam? IM1-7: Her cardiovascular exam was completely normal
Teaching around the case	Supervisors teach the team using a variety of teaching styles	Case 2; PM SR-6: So what’s the best route to replace potassium? CC-4: Orally. SR-6: Yeah, orally. Do you know why?
Thinking out loud	Supervisors convey their thoughts or provide their interpretation of the case	Case 19; AM A‑10: And common things being common, I mean, that probably was the trigger. It’d be highly unlikely that she’s got two independent things
Providing direction	Supervisors give instructions for managing the case	Case 14; AM A‑4: He’s going to need prolonged IV antibiotics, probably 6 weeks if he’s true osteo and someone’s going to need to follow that

*AM* indicates morning case presentation; *PM* overnight case presentation; *A* attending physician; *SR* senior resident; *IM1* first-year internal medicine resident; *FM1* first-year family medicine resident; *CC* clinical clerk

This is a nice example of how ‘Tab. 1’, conventionally used in quantitative research papers for demographic details of the research sample, can be re-conceptualized to feature the key findings from a qualitative analysis. Tables should be supplemented, however, with narrative explanation in which the writer contextualizes and interprets the quoted material. More on this in the section on Argument.

### Is the quote representative?

We have all been tempted to include the highly provocative quote (that thing we cannot believe someone said on tape), only to realize by the third draft that it misrepresents the data and must be relinquished. Quote selection should reflect strong patterns in the data; while discrepant examples serve an important purpose, their use should be purposeful and explicit. Your quote selection should also be distributed across participants, in order that you represent the data set. This may mean using the second- or third-best example rather than continuing to quote the same one or two highly articulate individuals.

You must provide sufficient context that readers can accurately infer the meaning of the quote. Sometimes this means including the interviewer’s question as well as the participant’s answer. In focus group research, where the emphasis is on the group discussion, it might be necessary to quote an exchange among participants rather than extracting individual comments. This example illustrates this technique:

*Interviewer: And, in your experience, how do the students respond to your feedback about how well they communicated?*

*SP1: Oh, really well, it’s really important to the students, they listen to what we say about their performance—*

*Interruption with overlapping talk*

*SP4: Well, yeah, on a good day maybe, sure. But not every time. Lots of sessions I feel like we’re probably more like props to them, so how well we think they did, I’m not sure that matters.*

*SP3: Don’t you find it depends on the student? (FG2)*

Of course, such a long excerpt threatens the goal of succinctness. Alternatively, you could use multiple quotes from this excerpt in a single sentence of your own:

*Some standardized patients in the group believed that their assessor role was ‘really important to the students, they listen to what we say about their performance’, while others argued that ‘we’re probably more like props to them, so how well we think they did, I’m not sure that matters’. (FG2)*

Sometimes a quote is representative but also, therefore, identifiable, jeopardizing confidentiality:

*One participant explained that, ‘as chair of the competency committee, I prioritize how we spend our time. So that we can pay sufficient attention to this 2nd year resident. She’s supposed to be back from maternity leave but she had complications so her rotations need some altering for her to manage.’ (CCC4, P2)*

In this case, the convention of using a legend (Clinical Competency Committee 4, participant 2) to attribute the quote may be insufficient to protect anonymity. If the study involves few programs and the methods identify them (e.g., Paediatrics and Medicine) and name the institution (e.g., Western University), the speaker may be identifiable to some readers, as may the resident.

## Argument

Quoted material does not stand on its own: we must incorporate it into our texts, both grammatically and rhetorically. Grammatical incorporation is relatively straightforward, with one main rule to keep in mind: quoted material is subject to the same sentence-level conventions for grammar and punctuation as non-quoted material. Read this example aloud:

*Arts and humanities teaching offers an opportunity for faculty to connect with medical students on a different level, ‘we can share how we feel about the work of caring, what it costs us, how it rewards us, as human beings’ (F9).*

Your ear likely hears that this should be two sentences. But quotation marks seem to distract us from this, and we create a run-on sentence by putting a comma between the sentences. An easy correction is to replace the comma with a colon.

*Arts and humanities teaching offers an opportunity for faculty to connect with medical students on a different level: ‘we can share how we feel about the work of caring, what it costs us, how it rewards us, as human beings’ (F9).*

Many writers rely on the colon as their default mechanism for integrating quoted material. However, while it is often grammatically accurate, it is not always rhetorically sufficient. That is, the colon doesn’t contextualize, it doesn’t interpret. Instead, it ‘drops’ the quote in and leaves the reader to infer how the quoted material illustrates or advances the argument. This is problematic because it does not fulfil the requirement for adequacy of interpretation in presenting qualitative results. As Morrow argues, writers should aim for a balance of their interpretations and supporting quotations: ‘an overemphasis on the researcher’s interpretations at the cost of participant quotes will leave the reader in doubt as to just where the interpretations came from; an excess of quotes will cause the reader to become lost in the morass of stories’ [4]. (p. 256).

There are many techniques for achieving this balance between researcher interpretations and supporting quotations. Some techniques retain the default colon but attend carefully to the material that precedes it. Consider the following examples:

*One clinician said: ‘Entrustment isn’t a decision, it’s a relationship’. (F21)*

*One clinician argued: ‘Entrustment isn’t a decision, it’s a relationship’. (F21)*

*One clinician in the focus group disagreed with the idea that entrustment was about deciding trainee progress: ‘Entrustment isn’t a decision, it’s a relationship’. (F21)*

*Focus group participants debated the meaning of entrustment. Many described it matter-of-factly as ‘the process we use to decide whether the trainee should progress’, while a few argued that ‘entrustment isn’t a decision, it’s a relationship’. (F21)*

These examples offer progressively more contextualization for the quote. The first example simply drops the quote in following the nondescript verb, ‘said’, offering no interpretive gloss and therefore exerting minimal rhetorical control over the reader. The second offers some context via the verb ‘argued’, which interprets the participant’s positioning or tone. The third interprets the meaning of the quote even more by situating it in the context of a focus group debate. And the fourth eschews the default colon entirely, integrating two quotes into the narrative structure of the author’s sentence to illustrate the dominant and the discrepant positions on entrustment in this focus group debate.

Integrating quotes into the narrative structure of your sentence, like the last example, offers two advantages to the writer. First, it interprets the quote for the reader and therefore exerts strong rhetorical control over the quote’s meaning. Second, it offers variety and style. If your goal is compelling prose, variety and style should not be underestimated. We have all had the experience of reading Results sections that proceed robotically: point-colon-quote, point-colon-quote, point-colon-quote …. If only to make the reader’s experience more enjoyable, your revision process should involve converting some of these to integrated narration.

Notwithstanding the goal of succinctness, sometimes you will include a longer quote because it beautifully illustrates the point. However, a long quote may offer opportunities for readers to focus on images or phrases other than those you intended, therefore creating incoherence in the argument you are making about your results. To guard against this, you might try the ‘quotation sandwich’ technique [5] of both an introductory phrase that sets up the context of the quote and a summary statement following it emphasizing why you consider it important and what you are using it to illustrate.

Finally, how many quotes do you need to support your point? More is not necessarily better. One quote should be sufficient to illustrate your point. Some points in your argument may not require a quoted excerpt at all. Consider this example, in which the first sentence presents a finding that is not illustrated with a quotation:

*Residents described themselves as being always tired. However, their perceptions of the impact of their fatigue varied, from ‘not a factor in the care I provide’ (R8) to ‘absolutely killing me … I’m falling asleep at the bedside’ (R15).*

The finding that residents are always tired does not require illustration. It is readily understandable and will not surprise anyone; therefore, following it with the quote ‘I’m tired all the time’ (R2) will feel redundant. The second part of the finding, however, benefits from illustration to show the variety of perception regarding impact.

If you do use multiple quotes to illustrate a point in your argument, then you must establish the relations between them for the reader. You can do this between the quoted excerpts or after them, as modelled above with the four examples used to illustrate progressively stronger quote contextualization.

In conclusion, quotes can be the life’s blood of your qualitative research paper. However, they are the evidence, not the argument. They do not speak for themselves and readers cannot infer what you intend them to illustrate. The authenticity principle can help you select a quote that is illustrative, succinct and representative, while the argument principle can remind you to attend to the grammatical and the rhetorical aspects of integrating the quote into the story you are telling about your research.

## Footnotes

1 A third situation is beyond the scope of this piece: translating quoted material from another language into English. For careful consideration of this issue, please see Helmich et al. [6].
